# Integration of stress hyperglycemia ratio and c-reactive protein level improves postoperative acute kidney injury risk stratification after noncardiac surgery: A nested case-control study

**DOI:** 10.12669/pjms.42.6.16085

**Published:** 2026-06

**Authors:** Xunqi Huang, Jing Wang, Longxin Zhang, Huale Zhang, Zhaodong Liu

**Affiliations:** 1 Xunqi Huang, Department of Anesthesiology, Fujian Maternity and Child Health Hospital, College of Clinical Medicine for Obstetrics & Gynecology and Pediatrics, Fujian Medical University, Fuzhou, Fujian Province 350001, P.R. China; 2 Jing Wang, Department of Anesthesiology, Fujian Maternity and Child Health Hospital, College of Clinical Medicine for Obstetrics & Gynecology and Pediatrics, Fujian Medical University, Fuzhou, Fujian Province 350001, P.R. China; 3 Longxin Zhang, Department of Anesthesiology, Fujian Maternity and Child Health Hospital, College of Clinical Medicine for Obstetrics & Gynecology and Pediatrics, Fujian Medical University, Fuzhou, Fujian Province 350001, P.R. China; 4 Huale Zhang, Department of Obstetrics and Gynecology, Fujian Maternity and Child Health Hospital, College of Clinical Medicine for Obstetrics & Gynecology and Pediatrics, Fujian Medical University, Fuzhou, Fujian Province 350001, P.R. China; 5 Zhaodong Liu, Department of Obstetrics and Gynecology, Fujian Maternity and Child Health Hospital, College of Clinical Medicine for Obstetrics & Gynecology and Pediatrics, Fujian Medical University, Fuzhou, Fujian Province 350001, P.R. China

**Keywords:** Acute Kidney Injury, Case Control, C-Reactive Protein Level, International Surgical Outcomes Study and Perioperative Research, Stress Hyperglycemia Ratio

## Abstract

**Background and Objective:**

The stress hyperglycemia ratio (SHR) has emerged as a potential predictor of acute kidney injury (AKI). However, its role in postoperative AKI (PO-AKI) after noncardiac surgery and interaction with systemic inflammation remains unclear. This study aimed to assess whether combining SHR with high-sensitivity C-reactive protein (hs-CRP) improves risk stratification.

**Methodology:**

This nested case-control study used the Korean INSPIRE database (2011–2020) to match 269 PO-AKI cases 1:4 with 1003 controls by age, sex, body mass index, and baseline creatinine. Conditional logistic regression was used to estimate adjusted odds ratios (aORs). Nonlinear associations were assessed using restricted cubic splines. Model performance was evaluated using changes in the C-statistic (ΔC), net reclassification improvement (NRI), and integrated discrimination improvement (IDI).

**Results:**

SHR demonstrated a U-shaped association with PO-AKI. Compared with the middle quartiles (Q2–Q3), both low (aOR = 1.48; 95% CI, 1.03–2.12) and high SHR (aOR = 1.66; 95% CI, 1.18–2.35) were associated with increased risk. Elevated hs-CRP (≥1 mg/L) further increased PO-AKI risk across SHR categories. Incorporating SHR and hs-CRP improved model performance (ΔC = 0.019; 95% CI, 0.002–0.036; P = 0.016; NRI = 0.244; P < 0.001; IDI = 0.013; P < 0.001).

**Conclusion:**

SHR demonstrated a U-shaped association with the PO-AKI risk, and elevated hs-CRP enhances risk stratification. Combined assessment of metabolic and inflammatory markers may improve identification of high-risk patients, supporting the integration of both biomarkers for identifying high-risk patients for intensified perioperative management.

## INTRODUCTION

Postoperative acute kidney injury (AKI) affects 7.2%–18.5% of the over 300 million patients undergoing non-cardiac surgery globally each year,^1^–^3^ posing a substantial burden on healthcare systems.^4^ Clinical practitioners rely mostly on preoperative blood glucose levels to assess the renal injury risk;^5^ however, this approach fails to differentiate between stress-induced and chronic hyperglycemia. This limitation may partially explain the discrepant results observed regarding the renoprotective effects of intensive preoperative glycemic management.^6^ Moreover, the combined effect of preoperative inflammatory status and stress-induced hyperglycemia on postoperative AKI (PO-AKI) remains unclear, leading to imprecise identification of high-risk populations.

The stress hyperglycemia ratio (SHR),^7^ recognized as a sensitive indicator of stress-induced hyperglycemia, has been proposed to predict the AKI risk.^8^,^9^ However, studies have yielded conflicting results on whether a low SHR increases the renal injury risk. Gao et al. first reported a linear association between SHR and AKI in patients with diabetes mellitus,^10^ a finding corroborated by Li et al. in patients with chronic kidney disease.^11^ By contrast, numerous recent studies have identified U-shaped^12^,^13^ or J-shaped associations^14^ between SHR and the incidence of AKI. The association between preoperative (SHR) and acute kidney injury (AKI) following non-cardiac surgery remains unexplored; determining whether an abnormally low preoperative SHR increases the postoperative renal injury risk is of paramount importance for perioperative glycemic management.

Preoperative comorbidities and other stimuli may induce concomitant alterations in both high-sensitivity C-reactive protein (hs-CRP) and the stress hyperglycemia ratio (SHR). Notably, Zhang et al. recently identified a potential association between hs-CRP and stress-induced hyperglycemia that collectively contributes to adverse outcomes;^15^ however, whether this association affects the PO-AKI risk remains unknown. Moreover, whether combining inflammatory and hyperglycemic biomarkers enhances the predictive accuracy for extreme-risk subgroups is unclear. We used a nested case-control design to systematically validate the association curve between SHR and AKI after non-cardiac surgery, and to estimate the interaction between CRP and SHR. We also quantified the incremental predictive value of integrating these biomarkers.

## METHODOLOGY

We collected data for this study from the INSPIRE (International Surgical Outcomes Study and Perioperative Research) database (version 1.2) a comprehensive perioperative database maintained by academic institutions in South Korea (https://inspire.or.kr).^16^ The database includes detailed medical records of approximately 130,000 patients who underwent surgery under anesthesia between 2011 to 2020. It was accessed in March 2024. Data extraction was performed using PostgreSQL version 14.0, ensuring precision and accuracy. The author (Xunqi Huang) obtained authorized access to the database. We conducted a case-control study to estimate the association between the SHR and the PO-AKI risk after non-cardiac surgery.

### Inclusion Criteria:

The records included had complete preoperative measurements of core variables:

- Serum glucose,
- Creatinine, high-sensitivity C-reactive protein (hs-CRP),
- Glycated hemoglobin (HbA1c).

### Exclusion Criteria:

We excluded records with:

- Pre-existing renal impairment (eGFR <60 mL/min/1.73m^2^, dialysis dependence, or renal failure diagnosis).
- Scheduled surgical duration <1 hour.
- Missing data in >20% of the non-core covariates used for multivariable adjustment. In the end, we included 269 PO-AKI cases and 1003 matched non-PO-AKI controls (Table-I). We adhered to the Strengthening the Reporting of Observational Studies in Epidemiology (STROBE) guidelines to conduct the study.

**Table-I:** Characteristics of Study Participants.

Variables	Case Participants (n = 1003)	Control Participants (n = 269)	P-value
SHR, median (IQR)	0.9 (0.8, 1.0)	0.9 (0.8, 1.1)	0.225
***Demographics***			
Age (years), median (IQR)	65.0 (60.0, 75.0)	65.0 (60.0, 75.0)	0.33
Women, *n* (%)	610 (60.8)	158 (58.7)	0.535
BMI (kg/m2), Mean ± SD	24.4 ± 3.9	24.2 ± 4.6	0.373
***Comorbidities, n (%)***			
Diabetes mellitus	174 (17.3)	52 (19.3)	0.45
Hypertension	58 (3.10)	51 (2.73)	0.663
Liver disease	28 (2.8)	16 (5.9)	0.012
COPD	2 (0.11)	3 (0.16)	0.173
Kidney	21 (2.1)	11 (4.1)	0.063
Cancer	296 (29.5)	79 (29.4)	0.963
Charlson comorbidity index, n (%)			0.001
<2	789 (79.3)	185 (69)	
≥2	206 (20.7)	83 (31)	
***Surgical conditions***			
Emergency	78 (7.8)	46 (17.1)	< 0.001
Type of anesthesia, n (%)			0.837
General anesthesia	747 (74.5)	202 (75.1)	
Neuraxial anesthesia	256 (25.5)	67 (24.9)	
Surgery approach, n (%)			0.425
Endoscopic	141 (14.1)	43 (16)	
Open	862 (85.9)	226 (84)	
Types of surgery, n (%)			< 0.001
Thoracic	62 (6.2)	22 (8.2)	
General Surgery	233 (23.2)	98 (36.4)	
Neurosurgery	110 (11)	15 (5.6)	
Obstetric and gynecological	19 (1.9)	1 (0.4)	
Otolaryngology	14 (1.4)	10 (3.7)	
Orthopedic Surgery	565 (56.3)	123 (45.7)	
ASA classification, n (%)			0.134
< 3	743 (74.1)	187 (69.5)	
≧ 3	260 (25.9)	82 (30.5)	
Operation time (hour), median (IQR)	2.4 (1.5, 4.2)	2.4 (1.4, 4.4)	0.811
Intraoperative hypotension, n (%)	136 (13.6)	35 (13)	0.815
Intraoperative bradycardia, n (%)	145 (14.5)	37 (13.8)	0.07
Crystalloid volume administered (mL), Median (IQR)	450.0 (100.0, 800.0)	550.0 (250.0, 800.0)	0.005
Colloid infusion reception, n (%)	279 (28)	81 (30.3)	0.44
Blood products infusion reception, n (%)	675 (67.6)	144 (53.9)	< 0.001
Vasopressor, n (%)	306 (30.5)	95 (35.3)	0.132
***Medication history, n (%)***			
Insulin	114 (11.4)	56 (20.8)	< 0.001
ACEI	107 (10.7)	32 (11.9)	0.567
Loop diuretics	38 (3.8)	44 (16.4)	< 0.001
***Laboratory tests, median (IQR)***			
HbA1c (%)	6.0 (5.6, 6.6)	6.1 (5.6, 7.1)	0.228
Glucose (mmol/L)	115.0 (101.0, 138.0)	120.0 (101.0, 145.0)	0.115
hs-CRP	0.2 (0.0, 0.5)	0.3 (0.1, 1.6)	<0.001
Hemoglobin (g/dL)	12.9 (11.6, 14.2)	11.9 (10.1, 13.7)	< 0.001
WBC (109/L)	6.7 (5.6, 7.9)	6.7 (5.2, 8.4)	0.826
Creatinine (mg/dL)	0.8 (0.7, 1.0)	0.9 (0.7, 1.2)	0.726

***Abbreviations:*** ACEI, angiotensin-converting enzyme inhibitors; ASA, American Society of Anesthesiologists; BMI, body mass index; COPD, chronic obstructive pulmonary disease; HbA1c, glycated hemoglobin; hs-CRP, high-sensitivity C-reactive protein; IQR, interquartile range; PO-AKI, postoperative acute kidney injury; SHR, stress hyperglycemia ratio; WBC, white blood cell.

### Ethical considerations:

Since our study involved the analysis of cases in a third-party, anonymized, publicly available database that had institutional review board (IRB) approval (No. H-2210-078-1368), our institution’s IRB approval was considered exempt in April 2024.

### Data collection Exposure:

We extracted surgical and anesthesia-related variables, diagnoses, vital signs, and laboratory results from the clinical data repository. hs-CRP, ABG and HbA1c values were obtained from the earliest preoperative records available after hospital admission. The collected data were categorized into the following domains: Demographics (age, sex, body mass index, comorbidities [diabetes mellitus, chronic obstructive pulmonary disease, chronic kidney disease, hypertension, cancer, Charlson comorbidity index [CCI, categorized as <2 or ≥2]); types of surgery (endoscopic, general, orthopedic, neurosurgery, obstetric and gynecological, otolaryngology, thoracic]); types of anesthesia (general anesthesia, neuraxial anesthesia); ASA classification; operation time; intraoperative events (intraoperative hypotension, intraoperative bradycardia); fluid management (crystalloid volume, colloid infusion, blood products infusion); estimated blood loss; urine output; vasopressor use; medication history (insulin, angiotensin-converting enzyme inhibitors, loop diuretics); and, laboratory tests (HbA1c, glucose, hs-CRP, hemoglobin, white blood cell counts). SHR has been extensively used to assess stress-induced hyperglycemia.

We obtained the results of initial blood glucose and glycated hemoglobin measurements from the participants upon admission. SHR was calculated via the following formula: SHR = ABG / (28.7 × HbA1c (%) − 46.7). Based on the quartile distribution (designating the lowest quartile as low, the second and third quartiles as middle, and the highest quartile as high), we stratified the SHR into low, medium, and high tiers. In addition, we stratified hs-CRP levels into elevated (≥1 mg/L) and normal (<1 mg/L) categories based on a clinical cut-off value of 1 mg/L. We identified comorbidities using the International Classification of Diseases, 9^th^ or 10^th^ revision (ICD-9 or ICD-10) codes. These included: Chronic obstructive pulmonary disease (COPD), chronic kidney disease (CKD), Hypertension, Cancer, Diabetes mellitus (defined as either a preoperative diagnosis of diabetes or an HbA1c level >6.5%). The diagnosis of AKI adhered to the KDIGO criteria, which include a ≥ 0.3 mg/dL (or ≥ 26.5 µmol/L) increase in serum creatinine within 48 h, a rise in serum creatinine to 1.5 times the baseline level within 7 days, or the need for continuous renal replacement therapy after surgery.

### Sample-size considerations:

Because this investigation is a retrospective, nested case–control study within the INSPIRE database, the number of cases was determined by available events. To inform readers about the study’s statistical precision, we therefore conducted a posthoc power appraisal using standard case-control approximations under a twosided α = 0.05. We modeled the exposure as categorical (SHR quartiles), taking the control exposure prevalence for the extreme quartiles as p0 = 0.25, and used the observed sample sizes (ncases = 269, ncontrols = 1,003). Under these conservative assumptions the sample has approximately 90% power to detect an OR of 1.67 (lowest quartile vs mid quartiles) and = 98% power to detect an OR of 1.92 (highest quartile vs mid quartiles).

### Statistical analysis:

We performed all statistical analyses using R (version 4.4.2), with two-tailed p-values <0.05 considered statistically significant. We used descriptive statistics to characterize the study population. Continuous variables are presented as means ± standard deviations (SDs) if normally distributed, or as medians with interquartile ranges (IQRs) if non-normally distributed. Categorical variables are summarized as frequencies and percentages (n, %). We assessed differences between the case (PO-AKI, *n =* 269) and control (no AKI, *n =* 1003) groups for baseline characteristics using appropriate tests: independent samples *t*-tests or Mann-Whitney *U* tests for continuous variables, and Chi-square tests or Fisher’s exact tests for categorical variables, as applicable.

We applied conditional logistic regression models to quantify the associations between the stress hyperglycemia ratio (SHR) categories and the PO-AKI risk, accounting for the nested case-control design. Three hierarchical models were constructed: Model-1 presents the unadjusted association; Model-2 was adjusted for core demographic and comorbidity variables (age, sex, BMI, ASA classification, Charlson comorbidity index); and, Model-3 was further adjusted for an extensive set of potential confounders, including specific comorbidities (diabetes, hypertension, COPD, cancer), preoperative laboratory values (hemoglobin, eGFR, WBC count), medication history (ACE inhibitors, insulin, loop diuretics), and surgical factors (operation time, surgery approach, type of anesthesia, intraoperative blood product infusion, colloid infusion, intraoperative hypotension, intraoperative bradycardia). Results are reported as odds ratios (ORs) with corresponding 95% confidence intervals (CIs), using the medium SHR category as the reference group. We excluded variables with more than 10% of missing data from the analysis and imputed those with less than 10% of missing data using multiple imputations by chained equations (MICE) to ensure unbiased estimates.

We examined the nonlinear association between SHR and PO-AKI risk using conditional logistic regression with a restricted cubic spline for folate levels, including three knots, and adjusting for covariates in Model-3. Nonlinearity was estimated using a likelihood ratio test. We further adjusted for CRP and tested multiplicative interactions by adding product terms to Model-3^17^ to assess whether hs-CRP modified the association between SHR and PO-AKI. We defined elevated CRP as >1 mg/dL. We stratified preoperative SHRs (low/medium/high) and hs-CRP status (normal/elevated), generating six cross-classified groups. This categorical variable was incorporated into Model-3 using medium SHR and normal hs-CRP as the reference group.^18^

To quantify the improvement in predictive performance gained by incorporating the SHR and hs-CRP into the baseline risk model, we evaluated three key metrics: changes in the C-statistic (ΔC), the continuous net reclassification improvement (NRI), and the integrated discrimination improvement (IDI). The statistical significance for ΔC was assessed using the DeLong test, whereas the significances for the NRI and IDI were determined using bootstrap methods with 1,000 resamples. These analyses formally test whether the addition of SHR and hs-CRP provides statistically and clinically meaningful enhancement to the model’s ability to discriminate cases from non-cases and correctly reclassify the risk. We calculated two-sided *P* values and 95% confidence intervals (CIs) for statistical inference. All analyses were conducted using R version 4.3.2 (primary packages: survival, Epi, rcssci, interaction R, and mediation). We conducted the data analysis from May to August 2024.

### Sensitivity analysis:

We demonstrated the stability of the associations observed by re-including the 10% of cases previously excluded due to missing data and showing that the results remained consistent.

## RESULTS

**Fig.1:**
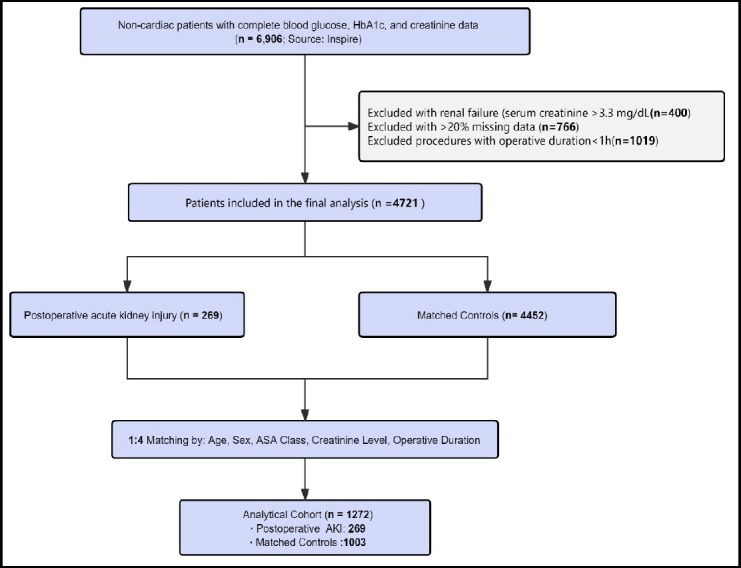
Flow chart.

We included data from 269 PO-AKI cases and 1003 matched controls (Table-I, Fig.1). Cases had significantly higher rates of emergency surgery (7.8% *vs* 17.1%, *P*<0.001) and intraoperative blood product transfusions (67.6% *vs* 53.9%, *P*<0.001). Preoperative use of insulin (11.4% *vs* 20.8%, *P*<0.001) and loop diuretics (3.8% *vs* 16.4%, *P*< 0.001) were also elevated in the cases. Laboratory findings revealed higher hs-CRP (0.3 *vs* 0.2 mg/L, *P*<0.001) and lower hemoglobin values (11.9 *vs* 12.9 g/dl, *P*<0.001) in cases, whereas the stress hyperglycemia ratios (SHRs) were similar in both groups (median 0.9, *P* = 0.225).

**Fig.2:**
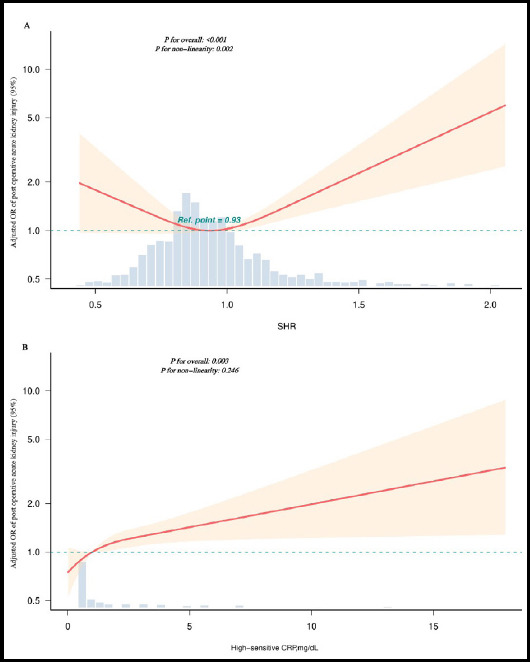
Restricted cubic spline.

We found a U-shaped association between the preoperative SHR and the PO-AKI risk (overall *P* <0 .001, *P*_for nonlinearity_ = 0.002) (Fig.2A). Notably, the optimal point in this curve, indicative of the lowest risk for all-cause mortality, was precisely identified at the specific SHR level of 0.93, aligning with the 0.801–1.04 interval we had designated as the reference group. In the unadjusted model (Model-1), both low (OR = 1.67; 95% CI, 1.18–2.36) and high (OR = 1.92; 95% CI, 1.38–2.65) SHR categories demonstrated significantly elevated PO-AKI risk compared to the medium reference category. Adjustment for core demographics and comorbidities in Model-2 modestly attenuated these associations, but both low (OR = 1.57; 95% CI, 1.11–2.22) and high (OR = 1.89; 95% CI, 1.36–2.62) SHR remained significant risk factors. Crucially, our comprehensive multivariable adjustment in Model-3 confirmed the persistent, independent associations of both the low (OR = 1.48; 95% CI, 1.03–2.12) and high (OR = 1.66; 95% CI, 1.18–2.35) SHR categories with an increased PO-AKI risk, exhibiting a U-shaped association (Table-II).

**Table-II:** Association between the stress hyperglycemia ratio (SHR) and the postoperative acute kidney injury (PO-AKI) risk.

Model	OR (95% CI)		
	Low	Medium	High
PO-AKI cases/controls, n/n	77/311	102/628	87/317
Model-1	1.67 (1.18–2.36)	1 [Reference]	1.92 (1.38–2.65)
Model-2	1.57 (1.11–2.2)	1 [Reference]	1.89 (1.36–2.62)
Model-3	1.48 (1.03–2.12)	1 [Reference]	1.66 (1.18–2.35)

Model-1: No adjustments, Model-2: age, sex, BMI, ASA, CCI2, Model-3: age, sex, BMI, ASA, CCI2, diabetes, hemoglobin, hypertension, COPD, cancer, ACEi, insulin, loop diuretics, eGFR, operation time, surgery approach, type of anesthesia, reception of blood products infusion, colloid infusion reception, WBC, intraoperative hypotension, intraoperative bradycardia.

***Abbreviations:*** ACEI angiotensin-converting enzyme inhibitors; CCI2, Charlson comorbidity index; CI, confidence interval; COPD, chronic obstructive pulmonary disease; eGFR, estimated glomerular filtration rate, hb; hemoglobin; OR, odds ratio; PO-AKI, postoperative acute kidney injury; SHR, stress hyperglycemia ratio; WBC, white blood cell.

We found an inverted L-shaped association between the preoperative hs-CRP levels and the PO-AKI (overall *P*<0.003, *P*_for nonlinearity_ = 0.246) (Fig.2B). Compared with the reference group (medium SHR/normal hs-CRP), the adjusted odds ratio (aOR) was 1.69 (95% CI, 1.13–2.51) for low SHR/normal hs-CRP, and surged to 4.48 (2.46–8.16) for low SHR/high hs-CRP. Elevated hs-CRP values accounted for 62.3% (95% CI, 41.5–79.8%) of the excess PO-AKI risk associated with a low SHR. Similarly, the high SHR/normal hs-CRP group exhibited an aOR of 1.99 (1.35–2.94), whereas this increased markedly to 4.03 (2.31–7.03) for high SHR/high hs-CRP. Systemic inflammation (hs-CRP ≥1 mg/L) amplified the PO-AKI risk across all SHR strata (Table-III).

**Table-III:** The Joint Association of SHR and CRP lever with PO-AIK Risk.

High-sensitive CRP	SHR		
	Low (Q1, <0.801)	Medium (Q2-Q3, 0.801, 1.043)	High (Q4, >1.043)
Normal^a^			
PO-AKI cases/controls, No./No.	53/250	72/518	58/241
aOR (95% CI)	1.69 (1.13~2.51)	1^c^	1.99 (1.35~2.94)
High^b^			
PO-AKI cases/controls, No./No.	24/61	30/110	29/76
OR (95% CI)	4.48 (2.46~8.16)	2.58 (1.53~4.35)	4.03 (2.31~7.03)
Multiplicative interaction^c^	*0.673*	NA	*0.491*

a: Normal levels defined as High-sensitive CRP below 1, b: High levels defined as High-sensitive CRP 1and above 1. c: Medium (Q2-Q3, 0.801, 1.043) SHR/normal CRP group served as the reference category against which the other SHR/hs-CRP combinations were compared. We used multiplicative interaction analyses with adjustment for age, sex, BMI, ASA, CCI2, diabetes, hb, hypertension, COPD, Cancer, Acei, Insulin, Loop diuretics, eGFR, Operation time, Surgery approach, Type of anesthesia, received blood products infusion, Received colloid infusion, Wbc, Intraoperative hypotension, Intraoperative bradycardia

***Abbreviations:*** aOR, adjusted odds ratio; NA, not applicable; Q, quantile.

**Fig.3:**
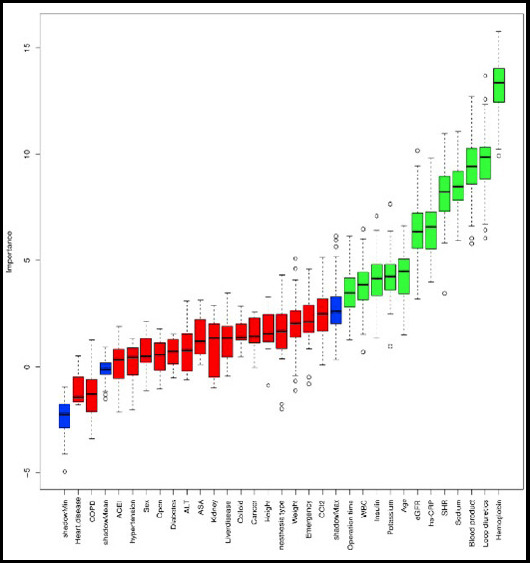
Boruta Tentative.

**Fig.4:**
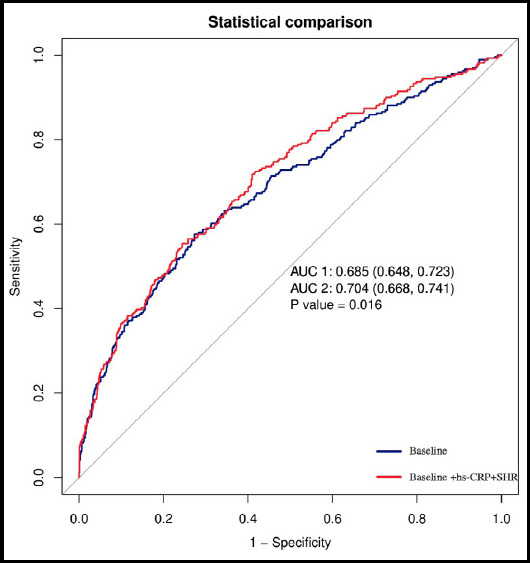
ROC curve comparison.

The feature selection results based on the Boruta algorithm are presented in Fig.3. We incorporated the variables identified as important, shown in the green area, into the development of the machine learning models. The addition of the SHR and hs-CRP values significantly enhanced the predictive capability of the baseline model for PO-AKI. The model C-statistic increased from 0.685 (95% CI, 0.648–0.723) to 0.704 (95% CI, 0.668–0.741), yielding a statistically significant ΔC of 0.019 (*P* = 0.016) (Fig.4). Further evidence of improvement was demonstrated by a significant continuous net reclassification improvement (NRI) of 0.244 (95% CI, 0.1102–0.3779; *P*<0.001), indicating substantially improved risk stratification. The integrated discrimination improvement (IDI) was also significant (IDI = 0.013; 95% CI, 0.0052–0.0208; *P*<0.001), confirming an overall increase in the model’s separation power between AKI and non-AKI patients (Table-IV).

**Table-IV:** Added predictive ability and reclassification statistics of the hs-CRP and the SHR as categorical variable.

	C-statistic (95% CI)	ΔC-statistic (95% CI)	P value	Continuous NRI (95% CI)	P value	IDI (95% CI)	P value
AKI							
*Baseline risk model^a^*	*0.685 (0.648,0.723)*	*Reference*		*Reference*		*Reference*	
Baseline risk model + hs-CRP + SHR	0.704 (0.668,0.741)	0.019	<0.016	0.244 (0.110-0.377)	< 0.001	0.013 (0.005-0.020)	< 0.001

CI confidence interval, hs-CRP High-sensitive CRP, IDI integrated discrimination improvement,

NRI net reclassification improvement, SHR stress hyperglycemia ratio.

a The baseline risk model comprised all variables selected by the Boruta algorithm, excluding SHR and hs-CRP.

## DISCUSSION

In this nested case-control study, we established a clinically significant U-shaped association between the preoperative Stress Hyperglycemia Ratio (SHR) and the postoperative acute kidney injury (PO-AKI) risk. Although we found no multiplicative interactions between the hs-CRP and SHR (*P*_for interaction_>0.05), the integration of both biomarkers substantially enhanced the model’s predictive capacity: an elevated hs-CRP amplified the PO-AKI risk at SHR extremes (particularly in cases with low SHRs), whereas our quantitative validation demonstrated a significant improvement in discrimination (ΔC = 0.019) and risk reclassification (NRI = 24.4%).

The SHR, initially conceptualized by Roberts et al.,^19^ has been substantiated by Gao’s research as a biomarker that effectively calibrates background glycemic levels and demonstrates superior prognostic utility over absolute hyperglycemia values in patients with critical illnesses.^10^ Preoperative SHR levels correlate significantly with major adverse cardiovascular and cerebrovascular events and mortality following noncardiac surgery, while enhancing the predictive accuracy for these endpoints.^20^ Although multiple studies have established associations between the SHR and AKI,^14^,^21^,^22^ few investigations on the association between preoperative SHR levels and the PO-AKI risk exist.

Perioperatve hyperglycemia is a prevalent phenomenon,^23^ yet considerable controversy persists regarding optimal glucose-lowering agents and glycemic targets. The SHR, integrating both baseline glycemia and acute stress-induced glucose levels, provides a novel perspective for perioperative glycemic management.^24^ Our results demonstrated a U-shaped association between the preoperative SHR and the noncardiac PO-AKI risk. These findings are consistent with emerging evidence demonstrating a nonlinear, often U-shaped relationship between SHR and adverse renal outcomes. A study by Li et al. reported a similar U-shaped association between the SHR and the in-hospital AKI risk among patients with heart failure.^12^ A recent report of Gao et al.^25^ also showed a nonlinear relationship between SHR and prognosis of patients with kidney injury. Such U-shaped correlation patterns extend beyond a mere statistical significance to offer crucial insights for refining individualized risk stratification and guiding targeted interventions.

The underlying mechanisms governing this U-shaped association remain incompletely elucidated. Surgical interventions constitute a potent physiological stressor, where maintaining an appropriate stress response is critical.^26^ Under profound surgical stress, moderate stress hyperglycemia may confer protective effects. Emerging evidence suggests that controlled glycemic elevation may safeguard cellular integrity via the resulting enhanced survival capacity and attenuated inflammatory damage.^27^ This phenomenon may stem from adaptive glucose metabolic reprogramming during moderate stress elevating the transcapillary glucose diffusion gradient.^28^ Such gradient maximization facilitates glucose uptake in microvascular-compromised tissues while upregulating prosurvival factors (*eg*, hypoxia-inducible factor-1α, vascular endothelial growth factor),^29^ thereby ensuring sufficient energetic substrates for immunocytes.^30^

Zhang et al. reported a significant multiplicative interaction between CRP and SHR for cardiovascular events in patients with myocardial infarction,^15^ whereas did not observe an interaction (*P*_for interaction_>0.05) between SHR and hs-CRP in patients undergoing noncardiac surgery. However, we found that elevated hs-CRP values substantially amplified the PO-AKI risk at both low and high SHR extremes (48% and 66% increased risk respectively *vs* normal hs-CRP; Table-III). Critically, incorporating both biomarkers significantly improved our model predictions: discrimination increased (ΔC = 0.019; 95% CI, 0.002–0.036; *P* = 0.016), risk reclassification improved (NRI = 0.244; 95% CI, 0.110–0.378; *P*<0.001), and the sensitivity was enhanced (IDI = 0.013; 95% CI, 0.005–0.021; *P*<0.001). This NRI magnitude (24.4% more accurate stratification) exceeds the minimal clinically important difference threshold (10%) for critical care. Existing prediction models for acute kidney injury after noncardiac surgery show good discrimination in the original population^31^ but have poor generalizability across different populations.^32^ The novel biomarkers identified in this study demonstrate favorable predictive ability and may offer an alternative for developing prediction models in other populations. The differential SHR-CRP patterns between surgical and cardiac cohorts suggest context-specific biological pathways. The hs-CRP-mediated risk amplification without a multiplicative interaction indicates distinct risk integration mechanisms in noncardiac surgery that warrant further investigation.

### Study Strength:

This study provides several novel contributions to the existing literature. While prior studies have examined the association between the SHR and AKI in specific populations, evidence regarding its role in predicting postoperative AKI in noncardiac surgery has been limited. Additionally, our findings reveal a U-shaped association between preoperative SHR and PO-AKI risk, extending beyond the predominantly linear or monotonic relationships that was reported before. Furthermore, by integrating SHR with an inflammatory marker (hs-CRP), we demonstrated a significant improvement in predictive performance and risk stratification, highlighting the potential value of combining metabolic and inflammatory indicators. Recent analyses in surgical cohorts have also shown that preoperative SHR is associated with adverse postoperative outcomes, further supporting its role as a perioperative risk marker.^33^ Together with previous reports, our findings have important clinical implications. SHR is a simple, readily available index that captures stress-induced glycemic imbalance, while hs-CRP reflects systemic inflammatory status. Their combined use may allow clinicians to better identify high-risk patients prior to surgery and guide individualized perioperative management strategies, including closer monitoring and optimization of metabolic and inflammatory conditions.

This study’s methodological strengths include the use of a large, well-characterized perioperative database; a nested case-control design with rigorous matching; comprehensive adjustment for confounding variables; and the application of advanced statistical approaches, including restricted cubic spline modeling and multiple metrics (ΔC, NRI, IDI) to evaluate predictive performance.

### Limitations:

It includes the exclusive preoperative measurement of glucose and hs-CRP levels precludes the evaluation of perioperative biomarker fluctuations. In addition, systemic inflammation was assessed using only hs-CRP, as other inflammatory biomarkers were not consistently available in the database.^34^ The use of a single marker may not fully capture the complexity of the inflammatory response, and future studies incorporating multiple inflammatory indicators are warranted to provide a more comprehensive assessment. Residual confounding may persist due to the observational nature of our study. Although our sample size is relatively large for perioperative data, the stratified analysis may lack sufficient power to adequately detect interactions. Finally, the case recruitment from a single Korean medical center may limit the extrapolation of our findings to diverse populations.

## CONCLUSIONS

This study demonstrated a U-shaped association between the preoperative stress hyperglycemia ratio (SHR) and the risk of postoperative acute kidney injury (PO-AKI), with this association further amplified by elevated high-sensitivity C-reactive protein (hs-CRP) levels. The significant improvement in the predictive model’s risk reclassification confirmed its clinical utility. Based on the results of this study, both biomarkers may be incorporated in the preoperative clinical testing for identifying high-risk patients requiring intensified perioperative management.

### Recommendations:

Further studies are needed to validate our findings in prospective, multicenter cohorts and across diverse populations. In addition, future research should explore the role of dynamic perioperative changes in SHR and inflammatory markers, as well as the integration of additional biomarkers, to further improve risk prediction and guide targeted interventions.

### Authors’ contributions:

**XH** and **HZ:** Literature search, study design and manuscript writing.

**LZ, XH** and **JW;** Data collection, data analysis and interpretation. Critical review.

**ZL:** Manuscript revision and validation and is responsible for the integrity of the study.

All authors have read and approved the final manuscript.
